# Analysis of the risk and risk factors for injury in people with and without dementia: a 14-year, retrospective, matched cohort study

**DOI:** 10.1186/s13195-018-0437-0

**Published:** 2018-10-30

**Authors:** Ruey Chen, Wu-Chien Chien, Ching-Chiu Kao, Chi-Hsiang Chung, Doresses Liu, Huei-Ling Chiu, Kuei-Ru Chou

**Affiliations:** 1 Department of Nursing, Taipei Medical University-Shuang Ho Hospital, No.291, Zhongzheng Rd., Zhonghe District, Taipei, 23561 Taiwan; 2 School of Nursing, College of Nursing, Taipei Medical University, No.250, Wu-Hsing Street, Taipei, 11031 Taiwan; 3 Department of Medical Research, Tri-Service General Hospital, No.325, Section 2, Cheng-Kung Road, Neihu District, Taipei, 11490 Taiwan; 4 Graduate Institute of Life Sciences, National Defense Medical Center, No.161, Section 6, Min-Chuan East Road, Neihu District, Taipei, 11490 Taiwan; 5 School of Public Health, National Defense Medical Center, No.161, Section 6, Min-Chuan East Road, Neihu District, Taipei, 11490 Taiwan; 6 Department of Nursing, Wan Fang Hospital, Taipei Medical University, No.111, Sec. 3, Xinglong Rd., Taipei, 11696 Taiwan; 7 Taiwanese Injury Prevention and Safety Promotion Association (TIPSPA), Taipei, Taiwan; 8 School of Gerontology Health Management, College of Nursing, Taipei Medical University, No.250, Wu-Hsing Street, Taipei, 11031 Taiwan; 9 Psychiatric Research Center, Taipei Medical University Hospital, No.252, Wuxing St, Xinyi District, Taipei, 110 Taiwan

**Keywords:** Injury, Dementia, Suffocation, Accidental drug poisoning, Falls, Suicide, Traffic, Abuse

## Abstract

**Background:**

Most previous studies on dementia and injuries have focused on a particular type of injury, and few studies have investigated overall injury in people with dementia. In this study, we investigated the risk factors and risk of overall injury, including the diagnosis, cause, and intentionality of injury, in people with and without dementia in Taiwan.

**Methods:**

We collected relevant data between 2000 and 2013 from the National Health Insurance Research Database (NHIRD). Overall, 455,630 cases, consisting of 91,126 people with dementia and 364,504 people without dementia, were included in this study and we performed subgroup analysis. A multivariate Cox proportional hazards regression analysis was used to determine the risk of injuries.

**Results:**

The 14-year follow-up data showed that people with dementia had a higher risk of injury-related hospitalization than did people without dementia (19.92% vs 18.86%, hazard ratio (HR) = 1.070, *p* < 0.001). Regarding the cause of injury, people with dementia were more likely to be hospitalized due to suffocation (HR = 2.301, *p* < 0.001), accidental drug poisoning (HR = 1.485, *p* < 0.001), or falls (HR = 1.076, *p* < 0.001), and were less likely to be hospitalized due to suicide or self-inflicted injury (HR = 0.670, *p* < 0.001) or a traffic accident (HR = 0.510, *p* < 0.001) than were people without dementia. Subgroup analysis showed that people with dementia with any of the three subtypes of dementia were at a higher risk of homicide or abuse than were people without dementia (vascular dementia, HR = 2.079, *p* < 0.001; Alzheimer’s disease, HR = 1.156, *p* < 0.001; other dementia, HR = 1.421, *p* < 0.001). The risk factors for overall injury included dementia diagnosis, female gender, age 65–74 years, and seeking medical attention for an injury within the past year.

**Conclusion:**

People with dementia are at a higher risk of injury-related hospitalization than people without dementia. The results of this study provide a reference for preventing suffocation, drug poisoning, and falls in people with dementia. In addition, government agencies should pay attention to and intervene in cases of abuse suffered by people with dementia.

## Background

There are now more than 47 million people with dementia worldwide, and this number is expected to rise to 131 million by 2050 [1]. Dementia comprises a group of symptoms that includes progressive decline in cognitive function, memory, language skills, spatial perception, computational capability, judgment and decision-making, abstract thinking, problem solving, attention, and even vision, balance, feeling and sensory skills, and motor skills [2–5]. Moreover, people with dementia may exhibit disturbing behavior, including sleep problems, paranoia/delusion, diurnal disturbance, aggressiveness, and personality changes; the condition can be so severe that it will interfere with their personal relationships and their ability to engage in daily activities [6–8].

Injury is a public health problem worldwide and has become a leading cause of death as lifestyles continue to evolve. Moreover, injury may cause long-term disability and severe trauma [9]. In Taiwan, according to the International Classification of Diseases, Ninth Revision, Clinical Modification (ICD-9-CM), injury is classified into three categories based on the following: 1) the nature of the injury: N-code (800–999), including fractures, dislocations, injuries, open wounds, contusions, crush injuries, burns, and poisoning, which facilitate clinical diagnosis and treatment; 2) the external cause of the injury: E-code (E800–E999), including traffic and transport accidents, food or drug poisoning, poisoning by other substances (solid, liquid, gas, vapor), fall, fire and flame, drowning and suffocation, suicide and self-injury, homicide, natural environment, accidents during medical procedures, and injury due to law enforcement or war; these causes of injury can help develop injury prevention programs; and 3) supplemental description: in case of N-code 800–999, an E-code (for external cause of injury) should be specified to indicate unintentional injury (E800–E949) or intentional injury (E950–E969) [10].

The cognitive function and performance of people with dementia declines over time, which may result in an increased risk of injuries. Therefore, it is essential to investigate the risk of injuries in people with dementia. However, most previous studies on dementia and injuries focused on a particular type of injury, such as suffocation, accidental falls, accidental drug poisoning, homicide or abuse, suicide and self-inflicted injury, and traffic accidents. Studies on suffocation showed that food infarctions were positively correlated with dementia and that people with dementia were prone to suffocation caused by foreign bodies [11, 12]. Moreover, among people with dementia, most cases of drug poisoning were accidental but were more severe than those in people without dementia [13, 14]. Studies showed that the risk of falls was 2–8 times higher in people with dementia than it was in healthy individuals [3, 15, 16]. Moreover, studies showed that 5% to 55% of people with dementia suffered homicide or abuse, whereas only 3.2% to 27.5% of general elderly individuals suffered abuse [17, 18]. For self-inflicted injury and traffic accidents, researchers have yet to reach an agreement. Recent publications indicate that people with dementia have a relatively high suicide rate in the early stages after dementia diagnosis [19, 20]; however, people with moderate to severe dementia have a decreased risk of suicide [21, 22]. People with dementia are 2–10 times more likely to die from traffic accidents than are people without dementia [4]; however, some studies have shown that people with dementia are at lower risk of traffic accidents than the general population [23]. These studies focused on a single type of injury and did not investigate overall injury or the risk factors for injuries in people with dementia. In this study, we investigated overall injury, including the diagnosis, cause, and intentionality of injury, and the risk factors for injuries in people with and without dementia.

## Methods

### Data sources

We collected relevant data from the National Health Insurance Research Database (NHIRD). National Health Insurance (NHI) was implemented in 1995 in Taiwan, and almost all residents are now covered (coverage rate: > 99%). The NHIRD is a database that was created for healthcare research institutes and includes information on the outpatient care, emergency treatment, and hospitalization of insured individuals. The diagnostic codes in the NHIRD are entered by physicians according to the ICD-9-CM. In this study each person with dementia was diagnosed by a neurologist or psychiatrist.

### Study design and sampled participants

**Fig. 1:**
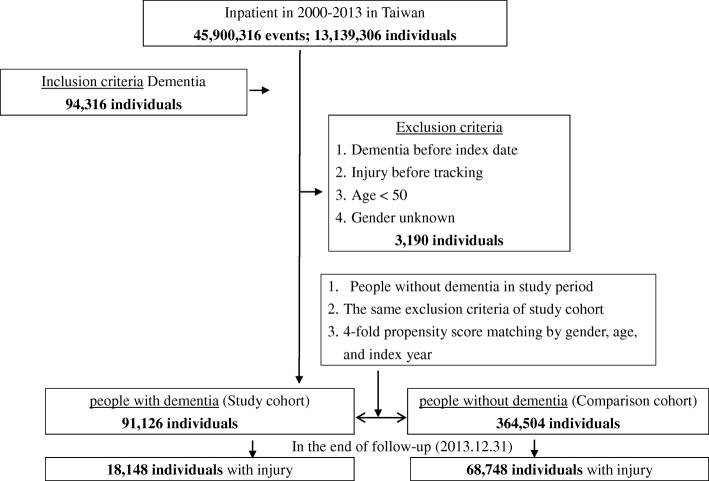
A flowchart of the study sample selection from the National Health Insurance Research Database in Taiwan

This was a retrospective, matched cohort study. Hospitalized people who had been diagnosed with dementia between 1 January 2000 and 31 December 2013 were included as three subtypes: 1) Alzheimer disease (i.e., senile dementia, presenile dementia, presenile dementia uncomplicated, presenile dementia with delirium, presenile dementia with delusional features, presenile dementia with depressive features, senile dementia with delusional or depressive features, senile dementia with delusional features, senile dementia with depressive features, senile dementia with delirium, and Alzheimer’s disease); 2) vascular dementia (i.e., arteriosclerotic dementia, arteriosclerotic dementia uncomplicated, arteriosclerotic dementia with delirium, arteriosclerotic dementia with delusional features, and arteriosclerotic dementia with depressive features); and 3) other dementias (i.e., other specified senile psychotic conditions, and unspecified senile psychotic conditions). People who had been diagnosed with dementia prior to 2000, had a history of injury, or were younger than 50 years of age were excluded from this study. Each person with dementia was matched with four people without dementia according to gender, age group, and index year. The covariates included gender and age group (Fig. 1). Since the data were pseudonymized on the basis of a pro-con analysis to protect people’s privacy, informed consent was waived. This retrospective analysis study was approved by the Joint Institutional Review Board of the Tri-Service General Hospital (TSGH IRB no. 1–105–05-142).

### Comorbidity

Baseline comorbidities included diabetes mellitus, hypertension, hyperlipidemia, cerebrovascular disease, chronic kidney disease, and alcohol-related disease.

### Outcome measures

The outcome measures were people who were admitted to hospital for injuries, including fractures, dislocations, sprains and strains, intracranial/internal injuries, open wounds, injury to blood vessels, superficial injuries/contusions, crush injuries, foreign body entering through an orifice, burns, injury to nerves and spinal cord, poisoning, and other injury, or coverage discontinuation.

### Statistical analysis

IBM SPSS v22 (IBM Corp., Armonk, NY, USA) was used for statistical analysis. Continuous variables are expressed as the mean ± standard deviation and analyzed with a *t* test; categorical variables are expressed as *n* (%) and analyzed with a Chi-square test. Multivariate Cox proportional hazards regression analysis was used to determine the risk of injuries, and the results are presented as hazard ratios (HRs) with 95% confidence intervals (CIs). The Kaplan-Meier method with a log-rank test was used to compare the risk of injuries between people with and without dementia. A *p* value < 0.05 was considered statistically significant.

## Results

### Characteristics of people with and without dementia

Table 1 shows that 455,630 individuals were included in this study, including 91,126 people with dementia—Alzheimer dementia 69,510 (76.28%), vascular dementia 1385 (1.52%), other dementia 20,231 (22.20%)—and 364,504 people without dementia. Of these, 237,945 (52.22%) were males, and 215,435 (47.28%) were aged 75 to 84 years. People with dementia had received more medical attention at clinics or emergency rooms in the last year for a previous injury than people without dementia (32.97% vs 30.76%, *p* < 0.001), and people with dementia had a higher percentage of cerebrovascular disease (13.33% vs 9.54%, *p* < 0.001) or alcohol-related disease (0.22% vs 0.16%, *p* < 0.001) than people without dementia. There were no significant differences between the groups in terms of gender and age distribution after propensity score matching.

**Table 1:** Characteristics of people with and without dementia

Variables	Total		People with dementia		People without dementia		*p* value
	*n*	%	*n*	%	*n*	%	
Total	455,630		91,126	20.00	364,504	80.00	
Gender							0.999
Male	237,945	52.22	47,589	52.22	190,356	52.22	
Female	217,685	47.78	43,537	47.78	174,148	47.78	
Age group (years)							0.999
50–64	21,660	4.75	4332	4.75	17,328	4.75	
65–74	85,675	18.80	17,135	18.80	68,540	18.80	
75–84	215,435	47.28	43,087	47.28	172,348	47.28	
≧85	132,860	29.16	26,572	29.16	106,288	29.16	
Catastrophic illness							< 0.001
Without	370,567	81.33	71,996	79.01	298,571	81.91	
With	85,063	18.67	19,130	20.99	65,933	18.09	
Injury outpatient/ER 1 year before index date							< 0.001
Without	313,474	68.80	61,083	67.03	252,391	69.24	
With	142,156	31.20	30,043	32.97	112,113	30.76	
Comorbidity							
Diabetes mellitus							< 0.001
Without	333,830	73.27	67,515	74.09	266,315	73.06	
With	121,800	26.73	23,611	25.91	98,189	26.94	
Hypertension							< 0.001
Without	280,288	61.52	58,684	64.40	221,604	60.80	
With	175,342	38.48	32,442	35.60	142,900	39.20	
Hyperlipidemia							< 0.001
Without	431,603	94.73	88,066	96.64	343,537	94.25	
With	24,027	5.27	3060	3.36	20,967	5.75	
Cardiovascular disease							< 0.001
Without	408,717	89.70	78,975	86.67	329,742	90.46	
With	46,913	10.30	12,151	13.33	34,762	9.54	
Chronic kidney disease							< 0.001
Without	412,668	90.57	85,668	94.01	327,000	89.71	
With	42,962	9.43	5458	5.99	37,504	10.29	
Alcohol-related disease							< 0.001
Without	454,861	99.83	90,925	99.78	363,936	99.84	
With	769	0.17	201	0.22	568	0.16	

Diabetes mellitus: International Classification of Diseases, Ninth Revision, Clinical Modification (ICD-9-CM) 250; hypertension, ICD-9-CM 401.1, 401.9, 402.10, 402.90, 404.10, 404.90, 405.1, 405.9; hyperlipidemia, ICD-9-CM 272; cerebrovascular disease, ICD-9-CM 433–434, 436; chronic kidney disease, ICD-9-CM 274.1, 403–404, 440.1, 442.1, 447.3, 572.4, 580–589, 642.1, 646.2, 753); alcohol-related disease, ICD-9-CM 291, 303

*p* value by Chi-square/Fisher exact test on category variables and *t* test on continue variables

*ER* emergency room

### Injuries and characteristics analysis

Table 2 shows the injury-related hospitalization rates. A higher percentage of people with dementia than people without dementia were hospitalized due to injury (19.92% vs 18.86%, *p* < 0.001). Further analysis of the diagnosis, cause, and intentionality of injuries showed that fracture was the most common diagnosis in both people with dementia and people without dementia (35.12% vs 38.49%); falls were the most common cause of injury in both people with dementia and people without dementia (50.15% vs 47.47%), followed by abnormal reactions to medical procedures (21.97% vs 23.79%) and other unintentional injuries (10.40%) in people with dementia and traffic accidents in people without dementia (11.55%). Unintentional injuries were most common in both people with dementia and people without dementia (98.90% vs 98.62%).

**Table 2:** Injury in people with and without dementia

Variables	Total		People with dementia		People without dementia		*p* value
	*n*	%	*n*	%	*n*	%	
Total	455,630		91,126	20.00	364,504	80.00	
Injury							< 0.001
Without	368,734	80.93	72,978	80.08	295,756	81.14	
With	86,896	19.07	18,148	19.92	68,748	18.86	
Injury diagnosis							< 0.001
Fracture	32,838	37.79	6374	35.12	26,464	38.49	
Dislocation	826	0.95	182	1.00	644	0.94	
Sprains and strains	1715	1.97	415	2.29	1300	1.89	
Intracranial/internal injury	9694	11.16	2246	12.38	7448	10.83	
Open wound	4504	5.18	1093	6.02	3411	4.96	
Injury to blood vessels	51	0.06	6	0.03	45	0.07	
Superficial injury/contusion	3494	4.02	826	4.55	2668	3.88	
Crushing	185	0.21	28	0.15	157	0.23	
Foreign body entering through orifice	1079	1.24	375	2.07	704	1.02	
Burn	936	1.08	228	1.26	708	1.03	
Injury to nerves and spinal cord	367	0.42	62	0.34	305	0.44	
Poisoning	3897	4.48	901	4.96	2996	4.36	
Others injury	27,310	31.43	5412	29.82	21,898	31.85	
Cause of injury							< 0.001
Traffic	5888	10.44	647	5.86	5241	11.55	
Poisoning (drugs/medicaments/biologicals)	816	1.45	212	1.92	604	1.33	
Poisoning (solid and liquid substances/gases/vapors)	191	0.34	41	0.37	150	0.33	
Surgical/medical care	137	0.24	30	0.27	107	0.24	
Abnormal reaction of medical procedures	13,221	23.44	2424	21.97	10,797	23.79	
Falls	27,075	48.00	5533	50.15	21,542	47.47	
Burns and fires	64	0.11	10	0.09	54	0.12	
Environment	216	0.38	33	0.30	183	0.40	
Drowning	8	0.01	1	0.01	7	0.02	
Suffocation	672	1.19	238	2.16	434	0.96	
Other unintentional injuries	4717	8.36	1147	10.40	3570	7.87	
Late effects	1324	2.35	314	2.85	1010	2.23	
Adverse drug reaction	1331	2.36	281	2.55	1050	2.31	
Suicide	580	1.03	82	0.74	498	1.10	
Homicide/abuse	168	0.30	39	0.35	129	0.28	
Intentionality of injury							0.018
Unintentional	55,660	98.67	10,911	98.90	44,749	98.62	
Intentional	748	1.33	121	1.10	627	1.38	

*p* value by Chi-square/Fisher exact test on category variables and *t* test on continue variables

**Fig. 2:**
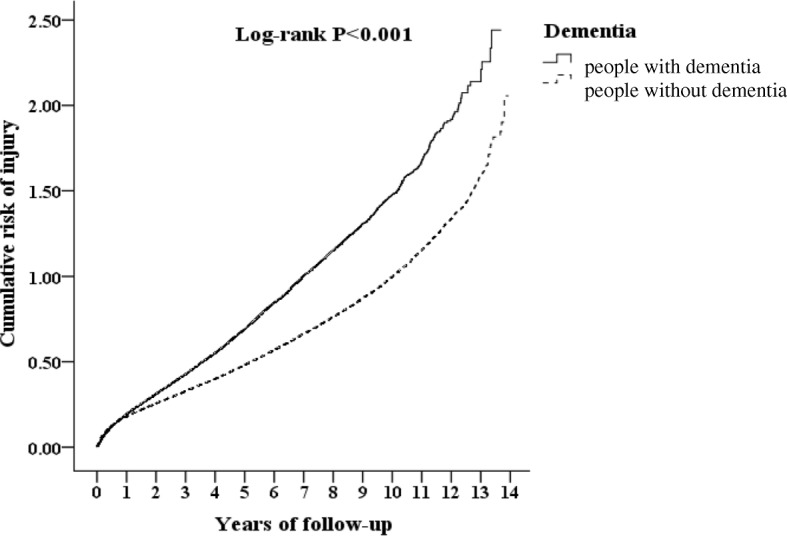
Kaplan-Meier curve of the cumulative risk of injury in patients aged 50 years and over stratified by dementia with log-rank test

The Kaplan-Meier analysis for the cumulative risk of injuries in individuals with and without dementia using a log-rank test showed a significant difference over the 14-year follow-up period (*p* < 0.001) (Fig. 2).

### Risk factors for overall injury

After controlling for comorbidities, Cox regression analysis showed that the risk factors for overall injury were dementia (HR = 1.070, *p* < 0.001), dementia subtype (Alzheimer’s disease, HR = 1.104, *p* < 0.001), female gender (HR = 1.100, *p* < 0.001), age 65–74 years (HR = 1.058, *p* < 0.001), and seeking medical attention for an injury at a clinic or emergency room within the last year (HR = 1.241, *p* < 0.001). Catastrophic illness (HR = 0.785, *p* < 0.001) was not a significant risk factor for injury-related hospitalization (Table 3).

**Table 3:** Risk factors for injury using Cox regression

Variables	Crude HR	95% CI	95% CI	*p* value	Adjusted HR	95% CI	95% CI	*p* value
Dementia								
People without dementia	Reference				Reference			
People with dementia	1.083	1.066	1.101	< 0.001	1.070	1.052	1.087	< 0.001
Dementia subtype								
Without	Reference				Reference			
Alzheimer disease	1.109	1.088	1.129	< 0.001	1.104	1.084	1.125	< 0.001
Vascular dementia	1.081	0.959	1.218	0.201	1.057	0.938	1.191	0.364
Other dementia	1.007	0.976	1.039	0.674	1.001	0.940	1.024	0.062
Gender								
Male	Reference				Reference			
Female	1.080	1.066	1.095	< 0.001	1.100	1.086	1.115	< 0.001
Age group (years)								
50–64	Reference				Reference			
65–74	1.050	1.021	1.079	0.001	1.058	1.029	1.088	< 0.001
75–84	0.877	0.856	0.899	< 0.001	0.864	0.843	0.886	< 0.001
≧85	0.658	0.641	0.675	< 0.001	0.630	0.614	0.647	< 0.001
Catastrophic illness								
Without	Reference				Reference			
With	0.788	0.775	0.802	< 0.001	0.785	0.771	0.799	< 0.001
Injury outpatient / ER 1 year before index date								
Without	Reference				Reference			
With	1.267	1.250	1.284	< 0.001	1.241	1.224	1.258	< 0.001

Adjusted for comorbidities (diabetes mellitus, hypertension, hyperlipidemia, cerebrovascular disease, chronic kidney disease, alcohol-related disease)

*CI* confidence interval, *ER* emergency room, *HR* hazard ratio

For the risk factors for injuries, further Cox regression analysis showed that people with dementia aged 50–64, 65–74, and 75–84 years were at an increased risk of injury-related hospitalization compared with people without dementia (HR 1.080-1.164) (Table 4).

**Table 4:** Factors for injury stratified by variables listed in the table using Cox regression

Stratified	People with dementia			People without dementia			Ratio	Adjusted HR	95% CI	*p* value
	Event	PYs	Rate (per 10^5^ PYs)	Event	PYs	Rate (per 10^5^ PYs)				
Total	18,148	142,971.64	12,693.43	68,748	596,593.59	11,523.42	1.102	1.070	1.052–1.087	< 0.001
Gender										
Male	9435	73,875.94	12,771.41	34,625	314,976.08	10,992.90	1.162	1.104	1.078–1.129	< 0.001
Female	8713	69,095.70	12,610.05	34,123	281,617.51	12,116.79	1.041	1.032	1.008–1.057	0.009
Age group (years)										
50–64	1531	9815.95	15,597.06	5860	43,124.98	13,588.41	1.148	1.164	1.100–1.232	< 0.001
65–74	3400	20,642.92	16,470.54	11,866	81,240.04	14,606.10	1.128	1.092	1.051–1.135	< 0.001
75–84	8185	59,944.66	13,654.26	31,122	255,921.18	12,160.78	1.123	1.080	1.054–1.107	< 0.001
≧85	5032	52,568.11	9572.34	19,900	216,307.39	9199.87	1.040	1.012	0.981–1.044	0.455
Catastrophic illness										
Without	14,846	111,309.27	13,337.61	56,572	471,309.04	12,003.16	1.111	1.065	1.045–1.084	< 0.001
With	3302	31,662.37	10,428.78	12,176	125,284.55	9718.68	1.073	1.118	1.076–1.163	< 0.001
Injury outpatient/ER 1 year before index date										
Without	10,585	51,095.49	20,716.11	41,104	209,544.31	19,615.90	1.056	1.055	1.033–1.078	< 0.001
With	7563	91,876.15	8231.73	27,644	387,049.28	7142.24	1.153	1.093	1.065–1.121	< 0.001

Adjusted for comorbidities (diabetes mellitus, hypertension, hyperlipidemia, cerebrovascular disease, chronic kidney disease, alcohol-related disease)

*CI* confidence interval, *ER* emergency room, *HR* hazard ratio, *PYs* person-years

### Injury type versus hospitalization risk

We analyzed the diagnosis, cause, and intentionality of injury. We also analyzed the hospitalization risks of different types of injury with Cox regression analysis. For the injury diagnosis, people with dementia were at a higher risk of hospitalization due to a foreign body entering through an orifice, sprains and strains, and burns than were people without dementia. For the cause of injury, people with dementia were at a higher risk of hospitalization due to suffocation and accidental poisoning by drugs. In contrast, people with dementia were at a lower risk of hospitalization due to suicide and self-inflicted injury and traffic accidents than were people without dementia. Also, for the intentionality of injury, people with dementia were at a higher risk of hospitalization due to unintentional injury than were people without dementia (Table 5). Subgroup analysis of three subtypes of dementia showed that people with dementia were at a higher risk of homicide or abuse than were people without dementia, regardless of dementia subtype, including vascular dementia (HR = 2.079, *p* < 0.001), Alzheimer’s disease (HR = 1.156, *p* < 0.001), and other dementia (HR = 1.421, *p* < 0.001).

**Table 5:** Factors for injury subgroup stratified using Cox regression

Dementia	People with dementia			People without dementia			Ratio	Adjusted HR	95% CI	*p* value
Injury subgroup	Event	PYs	Rate (per 10^5^ PYs)	Event	PYs	Rate (per 10^5^ PYs)				
Injury diagnosis										
Fracture	6374	142,971.64	4458.23	26,464	596,593.59	4435.85	1.005	0.992	0.965–1.020	0.573
Sprains and strains	415	142,971.64	290.27	1300	596,593.59	217.90	1.332	1.316	1.177–1.471	< 0.001
Intracranial/internal injury	2246	142,971.64	1570.94	7448	596,593.59	1248.42	1.258	1.204	1.148–1.263	< 0.001
Open wound	1093	142,971.64	764.49	3411	596,593.59	571.75	1.337	1.171	1.093–1.255	< 0.001
Foreign body entering through orifice	375	142,971.64	262.29	704	596,593.59	118.00	2.223	2.202	1.939–2.499	< 0.001
Burn	228	142,971.64	159.47	708	596,593.59	118.67	1.344	1.296	1.115–1.507	0.001
Poisoning	901	142,971.64	630.19	2996	596,593.59	502.18	1.255	1.206	1.118–1.300	< 0.001
Cause of injury										
Traffic	647	142,971.64	452.54	5241	596,593.59	878.49	0.515	0.510	0.470–0.553	< 0.001
Poisoning (drugs/medicaments/biologicals)	212	142,971.64	148.28	604	596,593.59	101.24	1.465	1.485	1.268–1.739	< 0.001
Falls	5533	142,971.64	3870.00	21,542	596,593.59	3610.83	1.072	1.076	1.044–1.108	< 0.001
Suffocation	238	142,971.64	166.47	434	596,593.59	72.75	2.288	2.301	1.961–2.701	< 0.001
Suicide	82	142,971.64	57.35	498	596,593.59	83.47	0.687	0.670	0.530–0.848	0.001
Homicide/abuse	39	142,971.64	27.28	129	596,593.59	21.62	1.262	1.107	0.770–1.590	0.584
Intentionality of injury										
Unintentional	10,911	142,971.64	7631.58	44,749	596,593.59	7500.75	1.017	1.018	1.007–1.040	0.009
Intentional	121	142,971.64	84.63	627	596,593.59	105.10	0.805	0.764	0.628–0.930	0.007

Adjusted for comorbidities (diabetes mellitus, hypertension, hyperlipidemia, cerebrovascular disease, chronic kidney disease, alcohol-related disease)

*CI* confidence interval, *ER* emergency room, *HR* hazard ratio, *PYs* person-years

## Discussion

In Taiwan, the recognition, management, and support services for people with dementia has been consistent and persistent from 2000 to 2018. In 2000, the government of Taiwan started its “long-term care” policy, the goal of which was to “develop a comprehensive long-term caring system for people in need to receive services. Therefore, their independence and quality of life increased and their capability of keeping dignity and self-reliance held firm” [24]. This is still the current policy and the result of this study provides considerations and suggestions for the government of Taiwan’s future plans for the policy.

### Risk factors for overall injury

The risk factors for injury-related hospitalization were dementia, female gender, age 65–74 years, and seeking medical attention for an injury at a clinic or emergency room within the last year. Whilst the difference in risk of injury between people with and without dementia was statistically significant, it was a small difference being only 1.07 times higher. Previous studies showed that people with dementia were at a higher risk of injury-related hospitalization than were people without dementia [3, 25], and women were 1.1 times more likely than men to be hospitalized for an injury. Previous studies reached different conclusions on the role of gender in injury-related hospitalization. A study in Australia concluded that females with dementia were at a lower risk of injury-related hospitalization than were males [25], whereas females with dementia were at a higher risk of falls and unintentional drug poisoning than were males [3, 13]. This study showed that the age group of 65–74 years was associated with a 1.058-fold higher risk of injury-related hospitalization than the age group of 50–64 years, whereas the age group of 75 years or above was associated with a lower risk of injury-related hospitalization than the age group of 50–64 years. A retrospective study on people with dementia versus people without dementia in Australia showed that individuals aged 65 and above were at a higher risk of injuries than were those aged 50–64 years; in particular, those aged 85 and above were 1.43 times more likely to have an injury than those aged 50–64 years [25], in contrast to the findings of this study which may be a result of where the study subjects lived (country) and the ethnicity and lifestyle of the subjects. Moreover, the Australian study used the ICD-10-CM diagnostic codes, whereas this study used the ICD-9-CM codes. Further research is needed for a more in-depth analysis.

### Injury subtypes and hospitalization risk in people with and without dementia

#### Suffocation and foreign bodies

According to the results of the study, people with dementia were 2.301 times more likely to be admitted for suffocation than were people without dementia. Individuals aged 65 years and above were more likely to die of suffocation (mostly due to food) than were those in other age groups [11, 26]. Many studies have shown that the risk factors for food-related suffocation include liquid or semi-solid food, poor dentition, alcohol, sedatives, and antipsychotics [11, 26–29].

In addition, foreign bodies may cause suffocation. A Japanese study analyzed the ingestion of inedible substances by psychiatric hospitalized people and showed that people with dementia were at the greatest risk of ingesting foreign bodies, followed by people with schizophrenia. Most cases involved the unintentional ingestion of foreign bodies regardless of dementia subtype, such as Alzheimer’s disease and vascular dementia. The most common foreign bodies ingested were diapers and gauze, and this occurred in the morning and before meals [12, 30]. People with dementia were prone to unintentional ingestion due to cognitive impairment; ingestion of inedible substances was a symptom of hyperorality in people with dementia [12, 31, 32]. Another study showed that people with dementia tend to have swallowing problems. As a result, we need to prevent people with dementia from swallowing foreign bodies, enhance their oral exercise training, swallow therapy, and dietary modification, and monitor their sedatives and antipsychotics intake.

#### Accidental poisoning by drugs, medicinal substances, and biologicals

People with dementia were 1.485 times more likely to be hospitalized for unintentional drug poisoning than were people without dementia, which was consistent with the findings of the Australian study [13]. The causes of unintentional overdose in elderly populations were the regular use of two or more medications, over-the-counter drugs or supplements, drug interactions, incorrect route of administration, no monitoring of drug concentrations, lack of knowledge about drugs, improper drug storage, sharing drugs with others, and adverse drug reactions [33–36]. People with dementia may have compliance problems, including how to take drugs as directed, and poor knowledge about drugs, or they may be unable to notice or handle adverse drug reactions, may mistake inedible substances (such as detergents) as drugs, and may take drugs via an incorrect route of administration [14, 35] because of declining memory, computational capability, judgment, and attention [2], resulting in an increased risk of hospitalization due to unintentional drug poisoning. To minimize these risks, drugs should be stored properly (drugs should be kept away from other similar-looking substances, and it should be ensured that the packaging is easy to identify and not broken), and a caregiver should become involved to help manage the medications [13, 33, 34] and monitor their adverse drug reactions.

#### Accidental falls

People with dementia were 1.076 times more likely to be hospitalized for accidental falls than were people without dementia. The recent literature and systematic reviews suggest that people with dementia are 2–8 times more likely to fall than are healthy individuals, with the former having an incidence of approximately 60–80% per year [15, 16, 37–40]. A prospective case-control study showed that elderly populations with cognitive impairment were two times more likely to fall than were those without cognitive impairment [41]. Even people with mild cognitive impairment (MCI) were at a higher risk of falls [40] and were 1.72 times more likely to fall than were those without cognitive impairment [42].

The risk factors for falls in people with dementia include the following: 1) cognitive impairment since people with dementia experience neurological and cognitive changes in the early stage of dementia, which impairs performance (attention, planning, orientation) and then gait (pace, step, dynamic balance); 2) motor disorders, including gait and balance disorders, and muscle weakness; and 3) behavioral disorders, including wandering and aggressive behaviors. People with dementia are also at risk of falls because they can potentially misjudge environmental hazards, overestimate their own ability, or suffer memory impairment. Antipsychotics are usually prescribed to improve psychiatric and behavioral symptoms in people with behavioral disorders, but these drugs may increase the risk of falls; finally, the presence of other health problems (such as orthostatic hypotension) can also increase the risk of falls [15, 40, 43–45]. As a result, it is crucial to understand the risk factors for falls in each individual person with dementia and provide much needed fall-prevention training.

#### Homicide or abuse

People with dementia along with any dementia subtype—vascular dementia, Alzheimer’s disease, and other dementia—were 1.156 to 2.079 times more likely to be hospitalized due to homicide or abuse than were people without dementia. Studies conducted in Amsterdam, the US, Ireland, London, Stockholm, and Kyoto showed that 5% to 55% of elderly people with dementia suffered abuse, which was significantly higher than the percentage observed in the general population (3.2–27.5%) [17, 18]. In the US, 500,000 to 2.5 million individuals aged 60 and above suffer abuse, especially at the hands of their spouse or children [46]. Abuse may be psychological, physical, or sexual [47], but the most common form is psychological abuse (~ 27.9–62.3%) [48]. Moreover, studies have shown that, for people with dementia, verbal abuse is most common (27–40%) [47], followed by physical abuse (3.5–23.1%) [48]. Unfortunately, we were unable to conduct a detailed analysis of abuse because the database contained no information on the different types of abuse.

People with dementia are more likely to suffer abuse than people without dementia for the following reasons: 1) confrontations between caregivers and people with dementia exhibiting psychological or behavioral symptoms, such as agitation and aggression; and 2) caregiver burden and stress [48]. For example, a survey in Australia surveyed homicidal ideation among primary caregivers of people with dementia and showed that 28.6% of primary caregivers actively or passively wished that the person would die [49]. Besides preventing the abuse of people with dementia, we need to acknowledge and provide support for the burden, stress, and emotional needs of the primary caregivers. This study may have underestimated the incidence rates of homicide or abuse because only severe homicide or abuse cases that resulted in hospitalization and were reported to government agencies were included in this study; cases where people with dementia did not seek medical attention or sought medical attention at clinics or emergency rooms were not reported. Thus, further research is needed to investigate this topic.

#### Suicide and self-inflicted injury

People with dementia were at a lower risk of suicide-related hospitalization than people without dementia, which may be a result of the cognitive and performance impairments of people with dementia.

Some researchers believe that people with dementia are at risk of suicide in the early stage of dementia, likely because they realize that they will become disabled and are concerned about the course of their disease and losing their independence. However, they still have the insight that their cognitive function will deteriorate and are concerned about the physical and economic burdens to their family; thus, they are at risk of suicide [19–21, 50]. As the disease progresses and insight and performance decline, people with moderate to severe dementia are less capable of preparing and planning a suicide, which reduces the risk of suicide [21, 22].

#### Traffic accidents

People with dementia were at a lower risk of traffic-related hospitalization than were people without dementia, which may be a result of the nature of the study subjects. The lower risk could possibly be due to the fact that a person with dementia may be less likely to go outside alone, without being accompanied by a family member or caregiver, compared with people without dementia. Hospitalized people with dementia may have been diagnosed with dementia for a long time, and their caregivers or healthcare providers would have reminded or asked them to stop driving, or those patients may no longer drive due to limited ability. A study in Australia showed that people with dementia were at a lower risk of traffic accidents within 3 years after hospitalization than were people without dementia (odds ratio 0.07), consistent with the findings of this study [5].

For people with dementia, cognitive impairment will affect vision, judgment, problem solving and decision-making skills, perception, and attention [4, 51, 52], which makes them prone to traffic accidents. However, recent studies have reached different conclusions. Some publications have shown that people with MCI or with dementia are 2–10 times more likely to die from traffic accidents while driving or riding motorcycles than are people without dementia [4, 53, 54]. However, some studies have shown no significant difference in the traffic accident rates between people with and without dementia [23, 55, 56]. Some studies reported that caregivers and healthcare providers asked people with dementia to stop driving for safety concerns [57, 58]. In addition, disease progression will severely affect memory, response, perception, the ability to perform simple tasks, and eventually the ability to drive; thus, people with dementia will stop driving 2 to 3 years after developing these symptoms [5, 59].

### Limitations

The present study possesses some limitations of note. The target population of this study were hospitalized people with and without dementia due to injury and who were aged 50 years and above. The result of this study cannot be generalized to people who are below the age of 50 years or outpatient and emergency patients. As a result, future research should further explore the risks and risk factors for people with and without dementia who are aged below 50 years and outpatient and emergency patients.

## Conclusion

The risk factors for injury-related hospitalization were dementia (especially Alzheimer’s disease), female gender, age 65–74 years, and seeking medical attention for an injury at a clinic or emergency room within the last year. For different types of injury, people with dementia were at a higher risk of hospitalization due to accidental drug poisoning, accidental falls, accidents caused by submersion, suffocation, and homicide or abuse than were people without dementia. The results of this study will serve as a reference for developing injury prevention and intervention programs for people with dementia, including guidance for caregiver assistance in drug management and how to prevent falls and suffocation caused by foreign bodies or food. In addition, government agencies should pay attention to the abuse suffered by people with dementia and should actively intervene and assist in handling related problems.

## Abbreviations

CI: Confidence interval
HR: Hazard ratio
ICD-9-CM: International Classification of Diseases, Ninth Revision, Clinical Modification
IRB: Institutional Review Board
MCI: Mild cognitive impairment
NHI: National Health Insurance
NHIRD: National Health Insurance Research Database
TSGH: Tri-Service General Hospital

## References

[1] Prince M, Comas-Herrera A, Knapp M, Guerchet M, Karagiannidou M. World Alzheimer Report 2016 Improving healthcare for people living with dementia coverage, Quality and costs now and in the future 2016.

[2] Cerejeira J, Lagarto L, Mukaetova-Ladinska EB. Behavioral and psychological symptoms of dementia. Front Neurol. 2012;3:73. 10.3389/fneur.2012.00073 PMC3345875 22586419

[3] Meuleners LB, Fraser ML, Bulsara MK, Chow K, Ng JQ (2016). Risk factors for recurrent injurious falls that require hospitalization for older adults with dementia: a population based study. BMC Neurol.

[4] Petersen JD, Siersma V, Nielsen CT, Vass M, Waldorff FB (2016). Dementia and traffic accidents: a Danish register-based cohort study. JMIR Res protocol.

[5] Meuleners LB, Ng J, Chow K, Stevenson M (2016). Motor vehicle crashes and dementia: a population-based study. J Am Geriatr Soc.

[6] Pinidbunjerdkool A, Saengwanitch S, Sithinamsuwan P (2014). Behavioral and psychological symptoms of dementia. J Med Assoc Thail.

[7] Ballard C, Corbett A, Chitramohan R, Aarsland D (2009). Management of agitation and aggression associated with Alzheimer's disease: controversies and possible solutions. Curr Opin Psychiatry.

[8] Lövheim H, Sandman P-O, Karlsson S, Gustafson Y (2008). Behavioral and psychological symptoms of dementia in relation to level of cognitive impairment. Int Psychogeriatr.

[9] Hofman K, Primack A, Keusch G, Hrynkow S (2005). Addressing the growing burden of trauma and injury in low-and middle-income countries. Am J Public Health.

[10] Statistics NCfH (2017). International classification of diseases, Ninth revision, clinical modification (ICD-9-CM).

[11] Kramarow E, Warner M, Chen L-H (2014). Food-related choking deaths among the elderly. Inj Prev.

[12] Yayama S, Tanimoto C, Suto S, Matoba K, Kajiwara T, Inoue M (2017). Analysis of inedible substance ingestion at a Japanese psychiatric hospital. Psychogeriatrics.

[13] Mitchell RJ, Harvey LA, Brodaty H, Draper B, Close JC (2015). Dementia and intentional and unintentional poisoning in older people: a 10 year review of hospitalization records in New South Wales, Australia. Int Psychogeriatr.

[14] Woolf A, Fish S, Azzara C, Dean D (1990). Serious poisonings among older adults: a study of hospitalization and mortality rates in Massachusetts 1983–85. Am J Public Health.

[15] Härlein J, Dassen T, Halfens RJ, Heinze C (2009). Fall risk factors in older people with dementia or cognitive impairment: a systematic review. J Adv Nurs.

[16] Allan LM, Ballard CG, Rowan EN, Kenny RA (2009). Incidence and prediction of falls in dementia: a prospective study in older people. PLoS One.

[17] Yan E, Kwok T (2011). Abuse of older Chinese with dementia by family caregivers: an inquiry into the role of caregiver burden. Int J Geriatr Psychiatry.

[18] Cooper C, Selwood A, Livingston G (2008). The prevalence of elder abuse and neglect: a systematic review. Age Ageing.

[19] Erlangsen A, Zarit SH, Conwell Y (2008). Hospital-diagnosed dementia and suicide: a longitudinal study using prospective, nationwide register data. Am J Geriatr Psychiatry.

[20] Draper B, Peisah C, Snowdon J, Brodaty H. Early dementia diagnosis and the risk of suicide and euthanasia: Elsevier; 2010;6(1):75–82. 10.1016/j.jalz.2009.04.1229 20129322

[21] Seyfried LS, Kales HC, Ignacio RV, Conwell Y, Valenstein M (2011). Predictors of suicide in patients with dementia. Alzheimers Dement.

[22] Cipriani G, Vedovello M, Lucetti C, Di Fiorino A, Nuti A (2013). Dementia and suicidal behavior. Aggress Violent Behav.

[23] Orriols L, Avalos-Fernandez M, Moore N, Philip P, Delorme B, Laumon B (2014). Long-term chronic diseases and crash responsibility: a record linkage study. Accid Anal Prev.

[24] Welfare MoHa (2018). Long-term care.

[25] Meuleners LB, Hobday MB (2017). A population-based study examining injury in older adults with and without dementia. J Am Geriatr Soc.

[26] Berzlanovich AM, Fazeny-Dörner B, Waldhoer T, Fasching P, Keil W (2005). Foreign body asphyxia: a preventable cause of death in the elderly. Am J Prev Med.

[27] Nikolić S, Živković V, Dragan B, Juković F (2011). Laryngeal choking on food and acute ethanol intoxication in adults—an autopsy study. J Forensic Sci.

[28] Dolkas L, Stanley C, Smith AM, Vilke GM (2007). Deaths associated with choking in San Diego county. J Forensic Sci.

[29] Inamasu J, Miyatake S, Tomioka H, Shirai T, Ishiyama M, Komagamine J (2010). Cardiac arrest due to food asphyxiation in adults: resuscitation profiles and outcomes. Resuscitation.

[30] Cullen P, Abid F, Patel A, Coope B, Ballard C (1997). Eating disorders in dementia. Int J Geriatr Psychiatry.

[31] Burns A, Jacoby R, Levy R (1990). Psychiatric phenomena in Alzheimer's disease. IV: disorders of behaviour. Br J Psychiatry.

[32] Ossenkoppele R, Pijnenburg YA, Perry DC, Cohn-Sheehy BI, Scheltens NM, Vogel JW (2015). The behavioural/dysexecutive variant of Alzheimer’s disease: clinical, neuroimaging and pathological features. Brain.

[33] Elliott RA (2006). Problems with medication use in the elderly: an Australian perspective. J Pharm Pract Res.

[34] Klein-Schwartz W, Oderda GM (1991). Poisoning in the elderly. Drugs Aging.

[35] Morgan TK, Williamson M, Pirotta M, Stewart K, Myers SP, Barnes J (2012). A national census of medicines use: a 24-hour snapshot of Australians aged 50 years and older. Med J Aust.

[36] Douglas A, Letts L, Richardson J (2011). A systematic review of accidental injury from fire, wandering and medication self-administration errors for older adults with and without dementia. Arch Gerontol Geriatr.

[37] Härlein J, Halfens RJ, Dassen T, Lahmann NA (2011). Falls in older hospital inpatients and the effect of cognitive impairment: a secondary analysis of prevalence studies. J Clin Nurs.

[38] Van Doorn C, Gruber-Baldini AL, Zimmerman S, Richard Hebel J, Port CL, Baumgarten M (2003). Dementia as a risk factor for falls and fall injuries among nursing home residents. J Am Geriatr Soc.

[39] Oliver D, Daly F, Martin FC, McMurdo ME (2004). Risk factors and risk assessment tools for falls in hospital in-patients: a systematic review. Age Ageing.

[40] Lach HW, Harrison BE, Phongphanngam S (2016). Falls and fall prevention in older adults with early-stage dementia: an integrative review. Res Gerontol Nurs.

[41] Taylor ME, Lord SR, Delbaere K, Mikolaizak AS, Close JC (2012). Physiological fall risk factors in cognitively impaired older people: a one-year prospective study. Dement Geriatr Cogn Disord.

[42] Delbaere K, Kochan NA, Close JC, Menant JC, Sturnieks DL, Brodaty H (2012). Mild cognitive impairment as a predictor of falls in community-dwelling older people. Am J Geriatr Psychiatry.

[43] Strubel D, Jacquot J, Martin-Hunyadi C (2001). Dementia and falls. Annales de readaptation et de medecine physique: revue scientifique de la Societe francaise de reeducation fonctionnelle de readaptation et de medecine physique.

[44] Shaw F (2007). Prevention of falls in older people with dementia. J Neural Transm.

[45] Sheridan PL, Hausdorff JM (2007). The role of higher-level cognitive function in gait: executive dysfunction contributes to fall risk in Alzheimer’s disease. Dement Geriatr Cogn Disord.

[46] Friedman LS, Avila S, Tanouye K, Joseph K (2011). A case-control study of severe physical abuse of older adults. J Am Geriatr Soc.

[47] Cooper C, Selwood A, Blanchard M, Walker Z, Blizard R, Livingston G (2009). Abuse of people with dementia by family carers: representative cross sectional survey. BMJ.

[48] Dong X, Chen R, Simon MA (2014). Elder abuse and dementia: a review of the research and health policy. Health Aff.

[49] O'Dwyer ST, Moyle W, Taylor T, Creese J, Zimmer-Gembeck MJ (2016). Homicidal ideation in family carers of people with dementia. Aging Ment Health.

[50] Lim WS, Rubin EH, Coats M, Morris JC (2005). Early-stage Alzheimer disease represents increased suicidal risk in relation to later stages. Alzheimer Dis Assoc Disord.

[51] Wagnera JT, Mürib RM, Nefc T, Mosimannc UP (2011). Cognition and driving in older persons. Risk.

[52] Taylor BD, Tripodes S (2001). The effects of driving cessation on the elderly with dementia and their caregivers. Accid Anal Prev.

[53] Friedland RP, Koss E, Kumar A, Gaine S, Metzler D, Haxby JV (1988). Motor vehicle crashes in dementia of the Alzheimer type. Ann Neurol.

[54] Tuokko H, Tallman K, Beattie BL, Cooper P, Weir J (1995). An examination of driving records in a dementia clinic. J Gerontol Ser B Psychol Sci Soc Sci.

[55] Man-Son-Hing M, Marshall SC, Molnar FJ, Wilson KG (2007). Systematic review of driving risk and the efficacy of compensatory strategies in persons with dementia. J Am Geriatr Soc.

[56] Trobe JD, Waller PF, Cook-Flannagan CA, Teshima SM, Bieliauskas LA (1996). Crashes and violations among drivers with Alzheimer disease. Arch Neurol.

[57] Seiler S, Schmidt H, Lechner A, Benke T, Sanin G, Ransmayr G (2012). Driving cessation and dementia: results of the prospective registry on dementia in Austria (PRODEM). PLoS One.

[58] Carr DB, O’Neill D (2015). Mobility and safety issues in drivers with dementia. Int Psychogeriatr.

[59] Gilley DW, Wilson RS, Bennett DA, Stebbins GT, Bernard BA, Whalen ME (1991). Cessation of driving and unsafe motor vehicle operation by dementia patients. Arch Intern Med.

